# The involvement of arbuscular mycorrhizal fungi in modulating polyamine metabolism and low-temperature tolerance enhancement in white clover

**DOI:** 10.3389/fpls.2025.1571852

**Published:** 2025-06-19

**Authors:** Songyuan Yao, Lin Li, Haitang Xiong, Peng Zhang, Tingting Ju, Zhiwei Chen, Lanlan Zheng

**Affiliations:** Shiyan Key Laboratory of Medicinal Plants and Evolutionary Genetics, School of Basic Medicine, Hubei University of Medicine, Shiyan, China

**Keywords:** arginine decarboxylase, chilling stress, chlorophyll, mycorrhiza, polyamine oxidase, putrescine

## Abstract

Sustained low temperatures can prevent white clover (*Trifolium repens* L.) from overwintering and regreening, making it difficult to revive the plants in the spring. Arbuscular mycorrhizal fungi (AMF) are widely known for their ability to enhance host stress tolerance. It is unclear whether AMF can enhance the low-temperature tolerance of white clover, which is associated with polyamines. The purpose of this study was to examine how inoculating white clover with an arbuscular mycorrhizal fungus *Funneliformis mosseae* affected the biomass, leaf chlorophyll and gas exchange, levels of root polyamines (putrescine, spermidine, and spermine), activities of key polyamine-related enzymes, and the expression level of the S-adenosyl-L-methionine decarboxylase (*TrSAMDC1*) gene at low temperatures (4°C for four days). The low-temperature treatment inhibited the root mycorrhizal colonization rate. Mycorrhizal inoculation significantly increased shoot, root, and total biomass, with greater increases found at optimal temperatures (22°C/18°C, 16 h/8 h, day/night temperature) than at low temperatures. Similarly, AMF inoculation significantly improved leaf gas exchange parameters, with larger increases observed at optimal temperatures than at low temperatures. Low temperatures caused a considerable increase in putrescine and spermidine levels, while simultaneously decreasing spermine levels. Mycorrhizal inoculation elevated putrescine, spermidine, and spermine levels regardless of temperature conditions, along with a significant rise in the (spermidine+spermine)/putrescine ratio. Mycorrhizal plants also exhibited considerably increased activities of arginine decarboxylase and polyamine oxidase, but not ornithine decarboxylase, in response to low temperatures. Mycorrhizal inoculation, together with low temperatures, elevated *TrSAMDC1* expression. The observed alterations in mycorrhiza-mediated polyamines were primarily attributed to increased arginine decarboxylase activity and *TrSAMDC1* expression. This study demonstrated the role of mycorrhizal fungi in modulating polyamine metabolism and enhancing plant tolerance to low-temperature stress.

## Introduction

White clover (*Trifolium repens* L.), a perennial herbaceous legume, is widely recognized for its high nutritional value, adaptability, and superior forage quality, making it a popular choice for ruminant feed (Han et al., 2007; Xie et al., 2021). In addition, it is frequently used with ryegrass to produce silage (Yan et al., 2023). Beyond its agricultural use, white clover is valued in horticulture and landscaping due to its early flowering, long green period, and high tolerance to trampling (Liang et al., 2021). White clover grows all across the world, predominantly in temperate and subtropical regions, where it flourishes in warm and humid climates, with optimal growth temperatures ranging from 19°C to 24°C (Yu, 2011). However, low temperatures are a major limiting factor in the development and use of white clover (Murray et al., 2000). Low temperatures have a noticeable negative impact on the growth, development, and metabolism of white clover plants, especially during winter (Yan et al., 2016). These adverse consequences appear as slower growth rates, lower nutrient levels, and poorer forage quality (Gusain et al., 2023). Sustained low temperatures can prevent white clover from overwintering and regreening, making it difficult to revive the plants in the spring (Murray et al., 2000). Such limitations not only reduce biomass yield, but also diminish the plant’s practical applications in agricultural and pastoral systems. Given these challenges, there is an urgent need to develop effective strategies to enhance white clover’s low-temperature tolerance.

Arbuscular mycorrhizal fungi (AMF) are a type of symbiotic fungi that live in soil and create symbiotic associations with the roots of most terrestrial plants (Smith and Read, 2008; Liu et al., 2024b). AMF develop arbuscules within the cortical cells of the host roots, allowing for the exchange of nutrients and carbon between the plant and the fungi (Liu et al., 2024a). Specifically, the host plant provides photosynthetic carbohydrates to the fungi, while the AMF absorb and transmit water and minerals (such as phosphorus, nitrogen, and potassium) from the soil and transfer them to the plant (Smith and Smith, 2011). As a result, AMF enhances the plant’s tolerance to both biotic and abiotic stresses, such as drought, low temperatures, salinity, pests, and disease (Shi et al., 2023). Earlier studies have demonstrated that AMF could impact plant responses to low-temperature circumstances (Tibbett and Cairney, 2007). AMF induced an increase in the activity of antioxidant enzymes (such as superoxide dismutase and catalase) in plants, thereby mitigating the oxidative damage caused by low temperatures (Liu et al., 2014). AMF can promote the accumulation of osmolytes in plants, such as proline and soluble sugars, allowing them to maintain intracellular water balance under low-temperature conditions (Lei et al., 2024). Furthermore, AMF can alter plant hormone levels (such as abscisic acid and auxins), regulating the plant’s adaptive responses to low temperatures (Wei et al., 2023). Under low-temperature conditions, AMF symbiosis has been shown to increase photosynthetic efficiency in plants at temperatures, alleviating the inhibition of photosynthesis (Li et al., 2020). However, AMF’s effects on plant low-temperature resistance are not uniformly constant. For example, in barley plants, AMF inoculation did not result in significant differences in growth variables (such as biomass and root length) and physiological indicators (such as photosynthetic efficiency and leaf water content) under low-temperature conditions when compared to uninoculated plants (Hajiboland et al., 2019). These findings indicate the complexities of AMF-mediated alteration of plant low-temperature resistance. Several studies have confirmed that AMF inoculation enhanced the drought resistance of white clover, a phenomenon linked to AMF-induced increases in nutrient uptake, accelerated accumulation of organic osmolytes, and enhanced antioxidant enzyme activities (Tuo et al., 2017; Liang et al., 2021). However, to date, no studies have confirmed whether AMF promotes the resistance of white clover to low-temperature stress.

Polyamines (PAs) are a type of low-molecular-weight aliphatic nitrogen-containing compounds prevalent in plants, animals, and microorganisms. The three main forms of PAs are putrescine (Put), spermidine (Spd), and spermine (Spm) (Kusano et al., 2008; Liang et al., 2022). PAs play a crucial role in fundamental cellular processes such as cell division, elongation, and differentiation processes, thereby promoting plant growth and development (Kusano et al., 2008). They can also affect gene expression and the gene regulatory networks of various physiological processes (Takahashi and Kakehi, 2010; Liu et al., 2015). During the biogenesis of photosynthetic apparatus, PA levels decreased (Dörnemann et al., 1996). Under stress conditions, one function of PAs is to prolong leaf senescence, specifically by inhibiting chlorophyll degradation and maintaining the structure of thylakoid membranes through their interaction with negatively charged sites on the membranes. This interaction helps preserve chlorophyll stability and functionality (Cohen et al., 1979; Cheng and Kao, 1983). Under adverse conditions, PAs stabilize the structure of thylakoid membranes, increase chlorophyll levels, and optimize the photosynthetic apparatus, thereby enhancing photosynthetic efficiency (Navakoudis et al., 2003; Alcázar et al., 2010). Related findings have been reported under various stress conditions, including salt stress, ozone stress, and drought stress (Demetriou et al., 2007; Zhao et al., 2024). Several studies have demonstrated that AMF promoted the synthesis of PAs, particularly Put, and accelerated the degradation of Spm and Spd to modulate physiology activities in response to drought and waterlogging stress (Zhang et al., 2020a; Zou et al., 2021; Liang et al., 2024). These findings suggest that PAs are involved in the regulation of plant stress resistance mediated by mycorrhizae. However, it remains unclear whether PAs are similarly involved in the low-temperature resistance of mycorrhizal plants and whether changes in PA levels are associated with photosynthetic physiology.

The objective of this study was to examine the effects of AMF inoculation on PA levels, PA-associated enzyme activity, biomass production, leaf chlorophyll and gas exchange parameters, and the expression levels of PA-associated genes in potted white clover under low-temperature stress. The study highlighted the potential of AMF as a bio-tool to improve the resilience of white clover under challenging environmental conditions, particularly in regions with fluctuating temperature regimes.

## Materials and methods

### Plant culture and experimental design

Prior to sowing, white clover seeds were surface-sterilized using 10% H_2_O_2_ for 10 min, followed by rinsing five times with sterile water and drying with filter paper to remove excess moisture. The growth substrate consisted of a 3:1 (v/v) mixture of soil and sand, which was autoclaved for 2 h prior to use. The nutrient content of the substrate was as follows: total nitrogen 653 mg/kg, ammonium nitrogen 1.78 mg/kg, nitrate nitrogen 37.48 mg/kg, and organic carbon 8.54 g/kg. Plastic pots with dimensions of 18 cm × 15 cm × 13 cm (upper diameter × lower diameter × height) were used. Prior to use, the pots were sterilized by wiping with 75% alcohol solutions. White clover seeds were sown on March 12, 2024, with 20 seeds per pot. These pots were placed in an environmentally controlled incubator with a temperature of 22°C during the day and 18°C at night (16/8 h photoperiod) (defined as optimal temperatures), 60% relative humidity, and a light intensity of 4,000 Lux. After 14 days, the seedlings were thinned to 13 plants per pot.

AMF inoculation was performed at the time of sowing. Based on the finding of Xie et al. (2020), the AMF strain used in this study was *Funneliformis mosseae* (T.H. Nicolson & Gerd.) C. Walker & A. Schüßler, provided by the Root Biology Institute of Yangtze University. The strain was propagated using maize as the host plant, and the AMF-infected root segments and growth substrate were collected to prepare the mycorrhizal fungal inoculum, which contained 25 spores/g. A total of 60 g of *F*. *mosseae* inoculum was applied to the designated pots (Xie et al., 2020). The inoculum was uniformly distributed at a depth of 8 cm above the bottom of the pots.

After 70 days of AMF inoculation, the plants were divided into two groups. One group was maintained under the original environmental conditions, while the other group was moved to a second growth incubator with a constant temperature of 4°C (day and night, 16/8 h photoperiod), along with other environmental conditions unchanged. The low-temperature treatment lasted for a period of 4 days, after which the plants were harvested to conclude the experiment.

The study consists of four distinct treatments: (i) inoculation with *F*. *mosseae* under optimal temperatures (OT+Fm), (ii) no inoculation with *F*. *mosseae* under optimal temperatures (OT-Fm), (iii) inoculation with *F*. *mosseae* under low temperature (LT+Fm), and (iv) no inoculation with *F*. *mosseae* under low temperature (LT-Fm). Each treatment was replicated six times, resulting in a total of 24 pots. The pots were arranged randomly within the growth chamber to minimize potential impacts of environmental heterogeneity.

### Measurement of root AMF colonization rate

The roots were stained for mycorrhizal assessment using the method described by Phillips and Hayman (1970). Briefly, root segments of 1 cm length (12 root segments per plant) were treated with 10% KOH solutions at 90°C until they became transparent. The root segments were then acidified in 1% HCl, rinsed four times, and stained in a 0.05% trypan blue-lactophenol solution at room temperature for 2 min. After staining, the root segments were destained in lactophenol solution. Root mycorrhizal colonization was observed under a microscope, and the mycorrhizal colonization rate was calculated as the percentage of the length of infected root segments relative to the total length of root segments.

### Measurement of plant growth and leaf gas exchange variables

The shoot and root parts of the plant were weighed after harvest to obtain shoot and root biomass. On the final day the experimental treatments, leaf gas exchange parameters, including net photosynthetic rate, transpiration rate, and stomatal conductance, were measured using a LI-6400 portable photosynthesis system. The measurement settings were as follows: the CO₂ concentration was 380 μmol/mol, the leaf chamber temperature was (25 ± 0.5) °C, and the airflow rate was set to 500 μmol/s. The light source was a built-in red-blue light source of the photosynthesis system, providing a photosynthetic photon flux density of 1000 μmol/m²/s. The measurement was taken on the fourth leaf from the top of the plant, with four replications per treatment.

### Measurement of PA levels in roots

The levels of Put, Spm, and Spd in roots were extracted following the protocol described by Liu et al. (2002). The 0.30 g of root sample was homogenized in 4 mL of pre-cooled 5% perchloric acid in an ice bath. After 1 h of ice bath extraction, the sample was centrifuged at 15,000 ×g/min for 30 min at 4°C. A 500 μL aliquot of the supernatant was incubated with 7 μL of benzoyl chloride, followed by the addition of 1 mL of 2 mol/L NaOH. The mixture was incubated at 37°C for 20 min. Next, 2 mL of saturated NaCl solution was added, and the mixture was extracted with 2 mL of diethyl ether. After centrifugation at 1,500×g/min for 5 min, 1 mL of the ether phase was collected and vacuum-dried. The dried residue was dissolved in 100 μL of methanol with vortexing and then filtered through a 0.45 μm membrane. Finally, 10 μL of the filtrate was injected into a high-performance liquid chromatography (HPLC) system (LC-100, Shanghai Wufeng Scientific Instrument Co., Ltd., Shanghai, China). The HPLC conditions were as follows: a C18 reverse-phase column (150 mm × 4.6 mm, 5 μm); mobile phase A, 0.1 mol/L ammonium acetate containing 0.1% acetic acid; mobile phase B, 0.1 mol/L ammonium acetate containing 0.1% acetic acid; the ratio of A to B with 60: 40; the injection volume, 20 μL; the flow rate, 0.8 mL/min; the column temperature, 35°C; and the UV detection wavelength, 254 nm.

### Measurement of PA-associated enzyme activities in roots

The activities of two polyamine synthetic enzymes (arginine decarboxylase, ADC; ornithine decarboxylase, ODC) and one polyamine catabolic enzyme (polyamine oxidase, PAO) were determined using Enzyme-Linked Immunosorbent Assay (ELISA) kits, with MC155L, ML058270, and ML092686 for the analysis, respectively. These kits were purchased from Shanghai Enzyme-linked Biotechnology Co., Ltd., Shanghai, China. The assays were conducted following the manufacturer’s instructions.

### Measurement of gene expression in roots

The expression of *S-adenosyl-L-methionine decarboxylase* (*TrSAMDC1*; GenBank accession: MN400662), a key gene in the polyamine synthesis pathway, was analyzed. Total RNA was extracted from the roots of each treatment group using the RNAprep Pure Plant Kit (TIANGEN). The integrity of the RNA was assessed using agarose gel electrophoresis, and its purity was verified by measuring the OD260/OD280 ratio. Qualified RNA was reverse transcribed into cDNA using the PrimeScript™ II 1^st^ Strand cDNA Synthesis Kit. The specific primers of *TrSAMDC1* were designed using Premier 5.0 software: forward primer, 5’-GCAAGCAAGTCATTTGAGCAG-3’; reverse primer, 5’-AATCAGAAGTCCCACCGTCG-3’. The *β-Actin* gene was used as the reference gene, with the following specific primer sequences for qRT-PCR: forward primer, 5’-TTACAATGAATTGCGTGTTG-3’; reverse primer, 5’-AGAGGACAGCCTGA ATGG-3’. qRT-PCR was performed using the SYBR Premix Ex Taq II kit, with the following amplification program: 95°C for 5 min; 95°C for 10 s, 57°C for 30 s, 40 cycles. Gene transcription levels were calculated using the 2^-ΔΔCt^ method (Livak and Schmittgen, 2001), with the OT-Fm treatment as the control.

### Statistical analysis

The experimental data were analyzed using SAS 8.0 software for analysis of variance (ANOVA). The significant differences between treatments were compared using Duncan’s New Multiple Range Test at a significance level of *p* < 0.05.

## Results

### Changes in root AMF colonization rate

**Figure 1:**
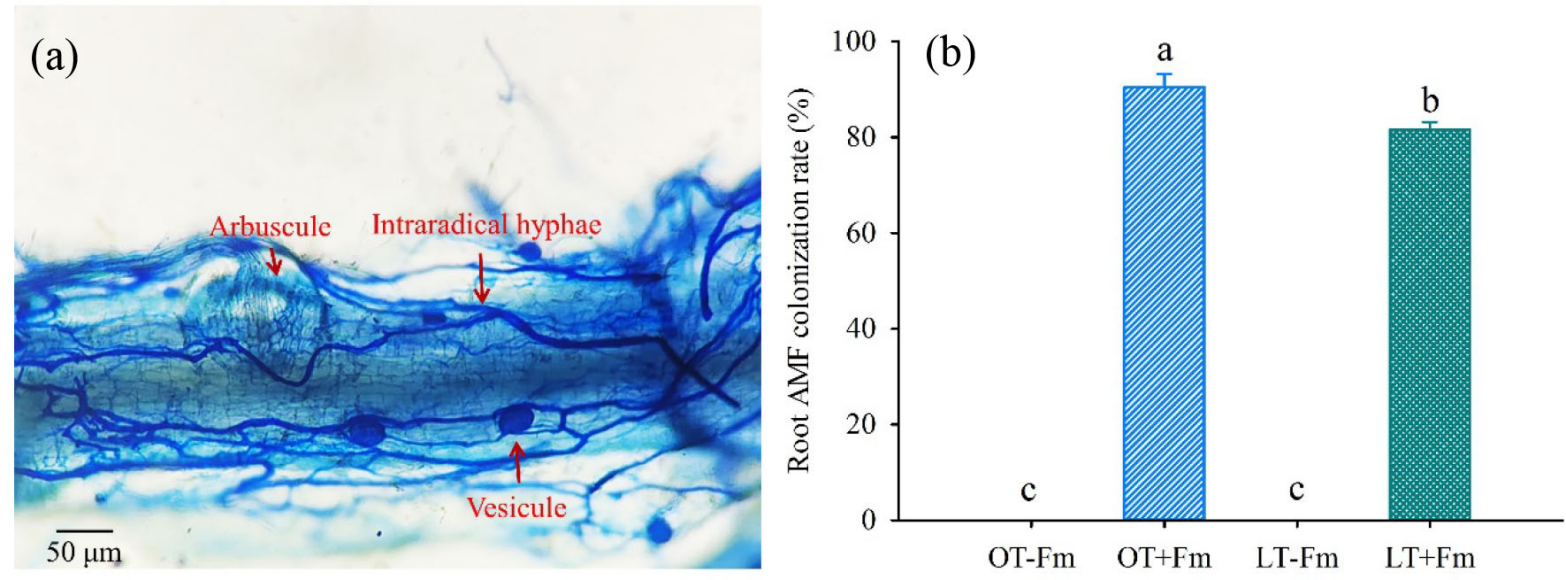
Root mycorrhizal colonization **(a)** and changes in root mycorrhizal colonization rate **(b)** of white clover by *Funneliformis mosseae* subjected to low-temperature treatment. Different letters above the bar (means ± SE, *n* = 5) indicated significant (*p* < 0.05) differences among treatments. OT, optimal-temperature; LT, low-temperature; +Fm, inoculation with (*F*) *mosseae*; -Fm, no-inoculation with (*F*) *mosseae*.

Mycorrhizal colonization was clearly visible in the roots of inoculated plants (**Figure 1a**), while no colonization was detected in uninoculated plants. The mycorrhizal colonization rate in the inoculated roots ranged from 81.55% to 90.49% (**Figure 1b**). Compared with the optimal-temperature treatment, the low-temperature treatment significantly inhibited the mycorrhizal colonization rate, as evidenced by a significant decrease of 9.88%.

### Changes in plant biomass

**Figure 2:**
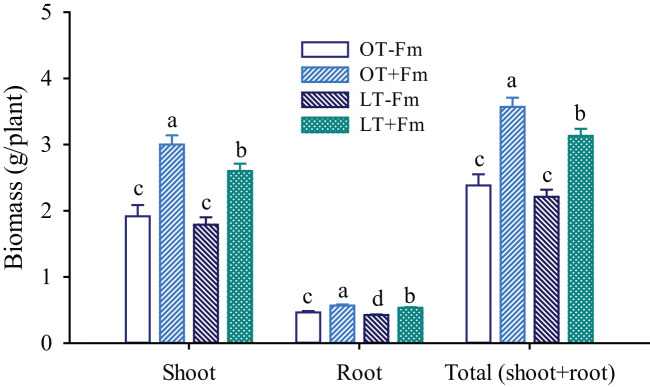
Changes in shoot, root, and total (shoot+root) biomass of white clover by *Funneliformis mosseae* subjected to low-temperature treatment. Different letters above the bar (means ± SE, *n* = 5) indicated significant (*p* < 0.05) differences among treatments. OT, optimal-temperature; LT, low-temperature; +Fm, inoculation with *F*. *mosseae*; -Fm, no-inoculation with *F*. *mosseae*.

The effects of the low-temperature treatment on biomass varied between inoculated and uninoculated plants (**Figure 2**). Specifically, compared to the optimal-temperature treatment, the low-temperature treatment only significantly inhibited the root biomass of no-AMF-inoculated plants by 8.36%, while it significantly inhibited the shoot, root, and total biomass of AMF-inoculated plants by 13.39%, 6.23%, and 12.25%, respectively. AMF inoculation significantly increased shoot, root, and total biomass, with increases of 56.42%, 22.41%, and 49.79% under optimal-temperature conditions, and 45.40%, 25.26%, and 41.53% under low-temperature conditions, respectively.

### Changes in SPAD value and gas exchange variables in leaves

Compared to the optimal-temperature treatment, the low-temperature treatment significantly inhibited the leaf SPAD value, net photosynthetic rate, transpiration rate, stomatal conductance, and intercellular CO_2_ concentration (**Table 1**). In uninoculated plants, these parameters decreased by 17.79%, 31.99%, 25.52%, 21.82%, and 5.26%, respectively, while in inoculated plants, they decreased by 15.22%, 22.81%, 21.63%, 20.02%, and 7.82%, respectively. Compared with no-AMF-inoculated plants, AMF-inoculated plants recorded significantly higher leaf SPAD values, net photosynthetic rates, transpiration rates, stomatal conductance, and intercellular CO_2_ concentrations, by 16.21%, 60.81%, 46.93%, 28.40%, and 12.58% under the optimal-temperature treatment, respectively, and by 19.85%, 56.12%, 40.66%, 24.51%, and 9.03% under the low-temperature treatment, respectively. In terms of the magnitude of increase, the effect of AMF on leaf gas exchange variables was more pronounced under optimal-temperature conditions than under low-temperature conditions.

**Table 1:** Changes in leaf SPAD value and gas exchange variables of white clover by *Funneliformis mosseae* subjected to low-temperature treatment.

Treatments	Net photosynthetic rate (μmol/m^2^/s)	Transpiration rate (mmol/m^2^/s)	Stomatal conductance (mmol/m^2^/s)	Intercellular CO_2_ concentration (μmol/mol)	SPAD value
OT-Fm	3.41 ± 0.14 c	3.13 ± 0.07 c	0.134 ± 0.006 b	270.2 ± 9.0 b	35.66 ± 1.25 b
OT+Fm	5.49 ± 0.11 b	4.60 ± 0.10 a	0.172 ± 0.003 a	304.2 ± 6.4 a	41.45 ± 1.01 a
LT-Fm	2.32 ± 0.11 d	2.33 ± 0.08 d	0.105 ± 0.005 c	256.0 ± 6.4 c	29.32 ± 0.66 c
LT+Fm	4.24 ± 0.27 b	3.60 ± 0.15 b	0.137 ± 0.002 b	280.4 ± 8.8 b	35.14 ± 1.22 b

Different letters following the data (means ± SE, *n* = 5) indicated significant (*p* < 0.05) differences among treatments. OT, optimal-temperature; LT, low-temperature; +Fm, inoculation with *F*. *mosseae*; -Fm, no-inoculation with *F*. *mosseae*.

### Changes in PA levels in roots

**Figure 3:**
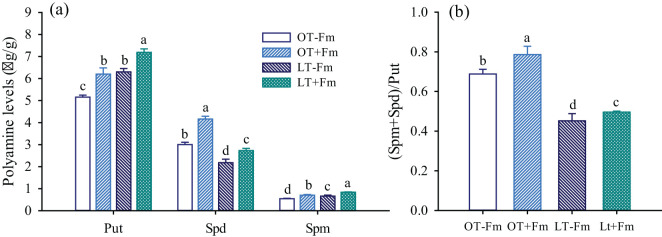
Changes in Put, Spd, and Spm levels **(a)** and (Spm+Spd)/Put ratio **(b)** of white clover roots by *Funneliformis mosseae* subjected to low-temperature treatment. Different letters above the bar (means ± SE, *n* = 5) indicated significant (*p* < 0.05) differences among treatments. OT, optimal-temperature; LT, low-temperature; +Fm, inoculation with (*F*) *mosseae*; -Fm, no-inoculation with (*F*) *mosseae*; Put, putrescine; Spd, spermidine; Spm, spermine.

In roots of white clover, Put was the predominant form among of PAs, followed by Spd and Spm (**Figure 3a**). Compared to the optimal-temperature treatment, the low-temperature treatment significantly increased the levels of Put and Spd in roots, by 22.15% and 21.98% in no-AMF-inoculated plants, and by 15.94% and 19.03% in AMF-inoculated plants, respectively. However, the Spd level in roots significantly decreased under low-temperature versus optimal-temperature conditions, with reductions of 27.34% and 34.45%, respectively. Under optimal-temperature conditions, AMF inoculation significantly increased the levels of Put, Spd, and Spm in roots by 20.21%, 38.61%, and 29.84%, respectively. Similarly, under low-temperature conditions, AMF inoculation also significantly increased the levels of Put, Spd, and Spm in roots by 14.10%, 25.04%, and 26.70%, respectively. Additionally, the low-temperature treatment significantly inhibited the (Spm+Spd)/Put ratio by 34.30% in no-AMF-inoculated plants and 36.90% in AMF-inoculated plants, respectively (**Figure 3b**). However, AMF inoculation significantly increased this ratio, by 14.36% and 9.82% under optimal and low temperatures, respectively, compared to the no-AMF treatment.

### Changes in PA-associated enzyme activities in roots

**Figure 4:**
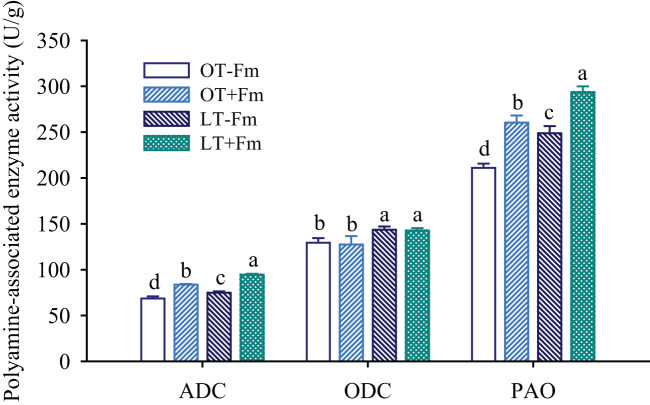
Changes in arginine decarboxylase (ADC), ornithine decarboxylase (ODC), and polyamine oxidase (PAO) activities of white clover roots by *Funneliformis mosseae* subjected to low-temperature treatment. Different letters above the bar (means ± SE, *n* = 5) indicated significant (*p* < 0.05) differences among treatments. OT, optimal-temperature; LT, low-temperature; +Fm, inoculation with *F*. *mosseae*; -Fm, no-inoculation with *F*. *mosseae*.

Compared to the optimal-temperature treatment, the low-temperature treatment led to an elevation in the activity of polyamine synthesis-related enzymes such as ADC and ODC in roots (**Figure 4**). Specifically, in no-AMF-inoculated plants, ADC and ODC activities increased by 9.26% and 10.96%, respectively, while in AMF-inoculated plants, they increased by 13.14% and 12.01%, respectively. AMF inoculation significantly increased the activity of ADC in roots, with increases of 21.98% and 26.32% under optimal and low-temperature conditions, respectively, compared to the no-AMF treatment. On the other hand, AMF treatment did not significantly affect the activity of ODC in roots, regardless of the temperature conditions. The activity of PAO, a PA catabolic enzyme, was also influenced by both AMF inoculation and low-temperature stress. Specifically, the PAO activity in roots of both uninoculated and inoculated plants significantly increased under low-temperature versus optimal-temperature, with rises of 17.91% and 12.79%, respectively. Additionally, AMF treatment further significantly increased the PAO activity in roots, with increases of 23.45% and 18.09% under optimal and low-temperature conditions, respectively.

### Changes in the relative expression of *TrSAMDC1* gene in roots

**Figure 5:**
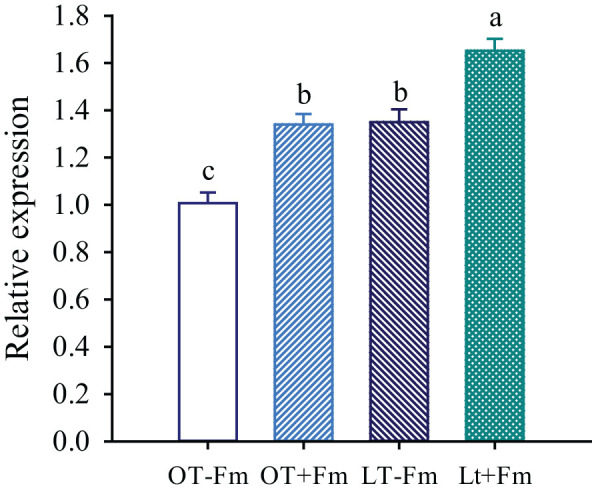
Changes in the relative expression of *S-adenosylmethionine decarboxylase* (*TrSAMDC1*) gene of white clover roots by *Funneliformis mosseae* subjected to low-temperature treatment. Different letters above the bar (means ± SE, *n* = 5) indicated significant (*p* < 0.05) differences among treatments. OT, optimal-temperature; LT, low-temperature; +Fm, inoculation with *F*. *mosseae*; -Fm, no-inoculation with *F*. *mosseae*.

The relative expression of the *TrSAMDC1* gene was significantly affected by both the low-temperature treatment and AMF inoculation (**Figure 5**). The low-temperature treatment significantly up-regulated the relative expression of the *TrSAMDC1* gene in roots of both no-AMF-inoculated and AMF-inoculated plants by 0.34- and 0.23-fold, respectively, compared to the optimal-temperature treatment. On the other hand, AMF inoculation significantly promoted the relative expression of the *TrSAMDC1* gene in roots, with increases of 0.33- and 0.22-fold under optimal-temperature and low-temperature conditions, respectively.

### Correlation analysis

Correlation analysis revealed significant (*P* < 0.01) positively correlations with the root colonization rate and root biomass, SPAD value, gas exchange variables, as well as root ADC, PAO, Put, Spd, and Spm. Among the PAs, Spd and Spm showed significantly positive correlations with root biomass, while only Spd was significantly positively correlated with leaf photosynthetic physiological variables. Additionally, Put was significantly positively correlated with *TrSAMDC1*, ADC, ODC, and PAO. Spd showed a significantly negative correlation only with ODC, while Spm was significantly positively correlated with *TrSAMDC1*, ADC, and PAO.

## Discussion

The present study indicated a relatively high root AMF colonization rate in *F*. *mosseae*-inoculated seedlings (**Figure 1b**), indicating a robust symbiotic relationship between the mycorrhizal fungus and the host plant. However, the root mycorrhizal colonization rate was significantly inhibited under low-temperature versus optimal-temperature conditions (**Figure 1b**), highlighted the sensitivity of *F*. *mosseae* to temperature fluctuations. This finding is in agreement with previous studies in barley, which demonstrated the adverse effects of low temperatures on mycorrhizal colonization and function (Li et al., 2020). The observed reduction in root AMF colonization rate under low-temperature conditions can be attributed to the reduced metabolic activity and growth of the mycorrhizal fungi, which impair their ability to form and maintain symbiotic structures within plant roots (Costa et al., 2013). As a result, temperature plays a critical role in regulating mycorrhizal colonization, with optimal-temperatures generally promoting colonization in some cases and low temperatures exerting an inhibitory effect (Rillig et al., 2002).

In no-AMF-inoculated plants, the low-temperature treatment had a significantly inhibitory effect on root biomass, not shoot and total biomass (**Figure 2**), indicating that the negative effect of low-temperature on white clover was more prominent in roots than in shoots. In contrast, AMF-inoculated plants exhibited a less pronounced reduction in shoot, root, and total biomass under low-temperature stress, suggesting that AMF-inoculated plants may allocate resources differently under low-temperature stress, potentially prioritizing root maintenance over shoot growth, which is critical for nutrient and water uptake during stress. The observed biomass reduction in mycorrhizal plants under low-temperature conditions could be attributed to the increased metabolic demands of mycorrhizas and the suppressed metabolic activity of cell division and elongation (Kiers and Heijden, 2006). Despite the negative impact of low temperatures on biomass in AMF-inoculated plants, AMF inoculation significantly increased shoot, root, and total biomass under both optimal and low-temperature conditions (**Figure 2**), suggesting the substantial benefits of mycorrhizal associations in promoting biomass accumulation under unfavorable temperature conditions. However, the magnitude of biomass increases under mycorrhization was lower under low-temperature conditions than under optimal-temperature conditions, suggesting that the stress still negatively affected the symbiotic relationship and plant growth (Ruiz-Lozano, 2003), even in the presence of AMF. On the other hand, significant differences in biomass production were already evident between AMF-inoculated and non-AMF-inoculated white clover plants prior to the low-temperature treatment. This pre-existing difference allowed AMF-inoculated plants to maintain their superior biomass levels under low-temperature stress conditions, contributing to their capacity to maintain elevated biomass during the stress period. The improved nutrient and water uptake, along with increased levels of Spd and Spm (**Table 2**), facilitated by AMF, likely played key contributors to the biomass enhancement (Sun et al., 2022; Liu et al., 2023). These findings suggest that AMF inoculation can mitigate the negative effects of low temperatures on plant biomass production, underscoring its potential as a tool for improving crop productivity in challenging environments, particularly in regions with variable temperature regimes (Asmelash et al., 2016). However, future studies should focus on monitoring changes in growth variable rates during the low-temperature treatment period, which can provide more robust evidence for the role of AMF by eliminating the influence of pre-existing growth differences between inoculated and non-inoculated plants prior to the stress treatment.

**Table 2:** The correlation coefficients among polyamines, photosynthetic physiological variables, mycorrhizal colonization rate, and root biomass.

	Root AMF colonization rate	Put	Spd	Spm
Root AMF colonization rate	1.00	0.61**	0.63**	0.74**
Root biomass	0.93**	0.37	0.79**	0.54*
SPAD value	0.69**	-0.08	0.93**	0.10
Net photosynthetic rate	0.88**	0.20	0.90**	0.37
Transpiration rate	0.86**	0.15	0.91**	0.33
Stomatal conductance	0.80**	0.15	0.85**	0.27
Intercellular CO_2_ concentration	0.77**	0.01	0.93**	0.17
*TrSAMDC1*	0.65**	0.94**	-0.13	0.95**
ADC	0.85**	0.88**	0.16	0.94**
ODC	-0.12	0.54*	-0.67**	0.43
PAO	0.75**	0.94**	0.01	0.94**

*P < 0.05; **P < 0.01.

In this study, low-temperature treatment significantly inhibits leaf SPAD values and several gas exchange parameters in both inoculated and uninoculated plants (**Table 1**), suggesting a decrease in chlorophyll synthesis or stability and photosynthetic efficiency, which can ultimately lead to reduced growth and productivity. However, AMF inoculation significantly enhanced leaf SPAD values and gas exchange parameters under both temperature conditions, with a higher magnitude of increase observed under optimal temperatures than under low temperatures (**Table 1**). These findings underscore the substantial role of mycorrhizas in enhancing leaf gas exchange and photosynthetic efficiency (Liu et al., 2022), although their effectiveness was somewhat diminished under low-temperature stress due to suppressed mycorrhizal colonization. AMF can enhance nutrient uptake, especially phosphorus, which is essential for ATP synthesis of and other components of photosynthetic machinery. This increased nutrient availability can lead to higher chlorophyll content (reflected in elevated SPAD values), improved photosynthetic efficiency, and increased stomatal activity. Nevertheless, at low temperatures, AMF activity in nutrient uptake and transfer to the plant may be reduced, and the plant’s ability to respond to AMF-mediated benefits may also be compromised (Gavito et al., 2003). Low temperatures can impair the metabolic activity of AMF hyphae, limiting their ability to explore the soil for nutrients and interact effectively with plant roots (Zhu et al., 2017). Correlation analysis showed that among the three PAs, only Spd showed a significantly positively correlated with SPAD values and leaf gas exchange variables (**Table 2**). Spd is known to improve photosynthetic efficiency by stabilizing the photosynthetic apparatus, enhancing chlorophyll synthesis, and protecting chloroplasts from oxidative damage (Alcázar et al., 2010). For instance, Spd has been shown to upregulate the expression of genes involved in chlorophyll biosynthesis and photosynthesis, such as *PtHEMA2* in citrus (Zhang et al., 2020b). In contrast, Spm is more associated with stabilizing cellular structures and membranes, which may not directly influence photosynthetic efficiency (Tiburcio et al., 2014). Put, as a precursor for the synthesis of Spd and Spm, primarily serves as a metabolic intermediate rather than a direct regulator of growth or stress responses.

Put is often the most abundant PA species due to its role as a precursor for the synthesis of Spd and Spm (Mustafavi et al., 2018), a phenomenon also observed in this study. Under stress conditions, such as low temperatures, PA levels often increase (Rhee et al., 2017), consistent with our findings of elevated Put and Spm levels in white clover roots exposed to low temperatures (**Figure 3a**). The relatively higher increase in Put and Spd levels in no-AMF-inoculated plants might indicate that AMF-inoculated plants possess additional stress-alleviating mechanisms (Wang et al., 2023). However, Spd levels in roots significantly decreased under low-temperature versus optimal-temperature conditions, likely due to its conversion to other PAs or degradation under stress conditions (Pál et al., 2021). AMF inoculation significantly increased the levels of Put, Spd, and Spm in roots of white clover, and root AMF colonization rate was significantly positively correlated with these PA levels (**Table 2**). Nevertheless, the magnitude of this increase was lower at low temperatures than at optimal temperatures (**Figure 3a**), indicating that the beneficial effects of mycorrhizae are partially constrained at low temperatures due to inhibited fungal growth and activity (Liu et al., 2004). In addition, it is possible that low temperatures limit host plant photosynthesis, resulting in less carbon being transferred to AMF, or that the carbon skeleton being used to produce PAs also decreases. Despite this, the increase in Put, Spd, and Spm levels under low-temperature conditions suggests that AMF inoculation enhances the plant’s stress response by modulating PA metabolism. Similar results have been observed in mycorrhizal peach under flooding conditions and mycorrhizal trifoliate orange under soil drought conditions (Zhang et al., 2020a; Zou et al., 2021; Liang et al., 2024). The higher levels of Spd and Spm, which are deemed to have stronger antioxidant properties than Put (Chen et al., 2018), may contribute to improved stress tolerance in inoculated plants. Further studies are needed to explore the interplay between AMF-modulated PAs and changes in antioxidant enzyme activities.

The low-temperature treatment significantly inhibited the (Spm+Spd)/Put ratio (**Figure 3b**), indicating a shift in the balance of PAs towards a higher proportion of Put, which is often associated with rapid stress responses. However, AMF inoculation significantly increased the (Spm+Spd)/Put ratio, suggesting a transformation of Put into Spm and Spd in a way (**Figure 3b**), which are more closely associated with long-term growth and development processes (Capell et al., 2004; Alcázar et al., 2010). The smaller increase in the (Spm+Spd)/Put ratio under low-temperature versus optimal-temperature conditions indicates that low-temperature stress partially limits the ability of AMF to restore PA balance to a more growth-promoting state (Groppa and Benavides, 2008).

The increase in the activity of polyamine synthesis-related enzymes (ADC and ODC) in roots under low-temperature treatment (**Figure 4**) was consistent with the observed increase in Put and Spd levels (**Figure 3a**). The higher increase in ADC activity in AMF-inoculated plants versus no-AMF-inoculated plants (**Figure 4**) may suggest that AMF further stimulates the plant’s Put-synthesis machinery in response to low temperatures. As a result, root Put levels were significantly positively correlated with root ADC activity. AMF inoculation significantly increased the activity of ADC in roots under both optimal and low-temperature conditions, conferring a mechanistic explanation for the AMF-mediated increase in PA levels. By enhancing ADC activity, AMF promoted the synthesis of Put, which can be further metabolized into other PAs. The lack of a significant effect of AMF on root ODC activity, as well as the absence of a significant correlation between ODC activity and root colonization rate, indicate that ADC may be a more critical target of AMF-mediated PA synthesis regulation. PAO, which is involved in the degradation of PAs (Hajiboland et al., 2019), showed increased activity in roots under AMF treatment and low-temperature stress (**Figure 4**). It suggests that AMF not only promotes PA synthesis but also influences their degradation, potentially modifying the PA pool to help plants adapt to varying environmental conditions.

In this study, the expression of the *TrSAMDC1* gene in roots was dramatically up-regulated by both low temperatures and AMF inoculation (**Figure 5**), along with a significantly positive correlation between *TrSAMDC1* expression and Spm levels. This up-regulation likely enhances the production of Spm, which is known to help plants cope with stress by stabilizing cell membranes, scavenging reactive oxygen species, and modulating gene expression (Blázquez, 2024). The more pronounced up-regulation of *TrSAMDC1* gene in no-AMF-inoculated plants versus AMF-inoculated plants suggests that AMF-inoculated plants may employ alternative stress-response mechanisms.

## Conclusions

In this study, low-temperature treatment exerted adverse impacts on biomass production, mycorrhizal colonization rate, leaf gas exchange, Spd, and the (Spm+Spd)/Put ratio in white clover plants. Nevertheless, AMF inoculation dramatically boosted biomass production, gas exchange, and PA (Spd, Spm, and Put) levels, with a more balanced PA composition towards an increased (Spm+Spd)/Put ratio. The AMF-mediated alterations in PAs were primarily attributed to increased ADC activity and enhanced expression of the *TrSAMDC1* gene, rather than ODC activity. These findings underscore the crucial role of mycorrhizal fungi in modulating PA metabolism and enhancing plant stress tolerance. However, this study was limited to examine the effects of a single arbuscular mycorrhizal fungal species under low-temperature conditions. Future research should broaden the scope by comparing multiple AMF species to provide a more comprehensive understanding of their roles in stress adaptation. Additionally, it is imperative to elucidate whether AMF-induced changes in host PAs are implicated in antioxidant defense responses, which could provide deeper insights into the complex mechanisms underlying plant-AMF interactions under stress conditions.

## Data Availability

The original contributions presented in the study are included in the article/supplementary material. Further inquiries can be directed to the corresponding author.
